# Skin Manifestations of Neuromyelitis Optica Spectrum Disorder With Secondary Systemic Lupus Erythematosus During Pregnancy: A Three-Year Follow-Up

**DOI:** 10.7759/cureus.40260

**Published:** 2023-06-11

**Authors:** Ikeoluwapo K Bolakale-Rufai, Ikechukwu Chukwuocha, Akintomiwa Makanjuola, Omololu Enigbokan, Joseph Yaria

**Affiliations:** 1 Clinical Translational Sciences, College of Medicine, University of Arizona, Tucson, USA; 2 Medicine, University College Hospital, Ibadan, NGA

**Keywords:** medical disorders in pregnancy, skin lesions, central nervous system disorder, optic nerve diseases, demyelinating autoimmune diseases

## Abstract

Neuromyelitis optica spectrum disorder (NMOSD) is a disease of the central nervous system and the optic nerves that disproportionately affects women and occasionally coexists with other autoimmune diseases. NMOSD manifesting as skin lesions is a rare phenomenon. Furthermore, these skin lesions in the setting of NMOSD during pregnancy have not been described. We report the case of a 31-year-old woman from sub-Saharan Africa who presented with initial recurrent skin lesions followed by paraparesis during her second trimester of pregnancy. Her next pregnancy was associated with sudden vision loss. She had positive serology for aquaporin-4 antibodies and subsequently developed a positive dsDNA antibody two years after the initial NMOSD diagnosis. Her skin lesions and symptoms improved following the administration of azathioprine. This case highlights the impact of pregnancy on NMOSD and the significance of a heightened level of suspicion for NMOSD in patients who exhibit recurring skin lesions preceding paraparesis events.

## Introduction

Neuromyelitis optica (NMO) is a disease that affects the central nervous system (CNS) and the optic nerves. Recent diagnostic criteria require the presence of optic neuritis, acute longitudinal myelitis, and other supporting criteria, which include seropositivity for aquaporin-4 antibodies (NMO IgG) [1]. Various pieces of evidence show inflammatory demyelination targeting the astrocytic water channel aquaporin-4 as the pathologic process for the abnormalities seen, with a preference for specific CNS regions [2]. The association of aquaporin-4 antibodies has led to the inclusion of other clinical manifestations in the diagnosis of this disease and a new nomenclature with a more inclusive term, neuromyelitis optica spectrum disorders (NMOSD). Various cases of NMOSD have been described, with reports highlighting clinical presentations, co-existing diseases, challenges in diagnosis, and treatment options [3]. Females of reproductive age are more likely to develop NMOSD, and recent reviews have also shown that pregnancy triggers disease onset, increases the relapse rate, and worsens disability [3-5]. However, skin lesions remain a rare and often overlooked manifestation of NMOSD. In this article, we report the skin manifestations of NMOSD in a pregnant woman who later developed systemic lupus erythematosus as an additional autoimmune comorbidity.

## Case presentation

**Figure 1:**
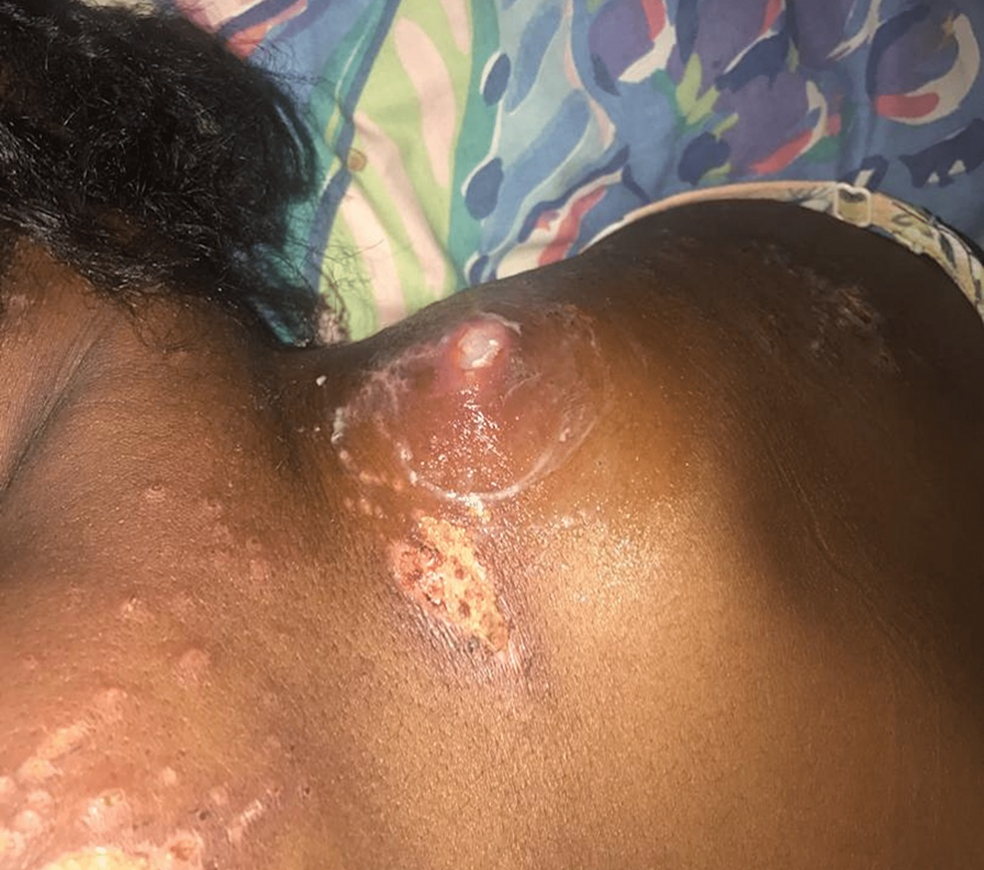
Active papular cutaneous lesions at the upper truncal area of the patient with NMOSD

The patient, a 31-year-old woman in her 23rd week of pregnancy, was admitted to the inpatient ward by the obstetricians on account of bilateral lower limb weakness and an "abnormal sensation" in her truncal area. Her complaints were preceded by generalized cutaneous lesions involving the trunk and extremities, noticed a few weeks after conception (Figure 1).

Initially presenting as intensely pruritic, hyperpigmented macular rashes, the lesions subsequently transformed into papular eruptions accompanied by serous discharge. These skin lesions involved the right trunk (T9-T10 dermatomes), the upper part of the chest (left side), and the popliteal surface of both lower limbs and the left wrist. There was no involvement of the palms or mucosal surfaces. She was prescribed medications, including opioids, chlorpheniramine, and prednisolone, but had no improvement in her symptoms. During her previous pregnancy, three years earlier, she disclosed experiencing bilateral paraparesis, which was preceded by similar cutaneous eruptions. However, at that time, she had a full resolution of symptoms postpartum with the delivery of a healthy neonate.

During her hospital stay, a neurology consult was scheduled, and she received intravenous antibiotics, oral antifungal medication, and tetanus prophylaxis. She was discharged home on prenatal vitamins without resolution of limb weakness. Neuroimaging, cutaneous biopsy, and further requested investigations were not conducted at the time due to financial implications. Despite her neurological deficits, she successfully had a vaginal delivery of a healthy newborn without any complications.

Four weeks after her postpartum wellness visit, she presented to the neurology clinic with acute visual impairment in her left eye, distressing flexor spasms, and sustained bilateral lower limb weakness. There was no history of fever, night sweats, weight loss, or coughing. A review of her past medical history revealed no additional information.

**Figure 2:**
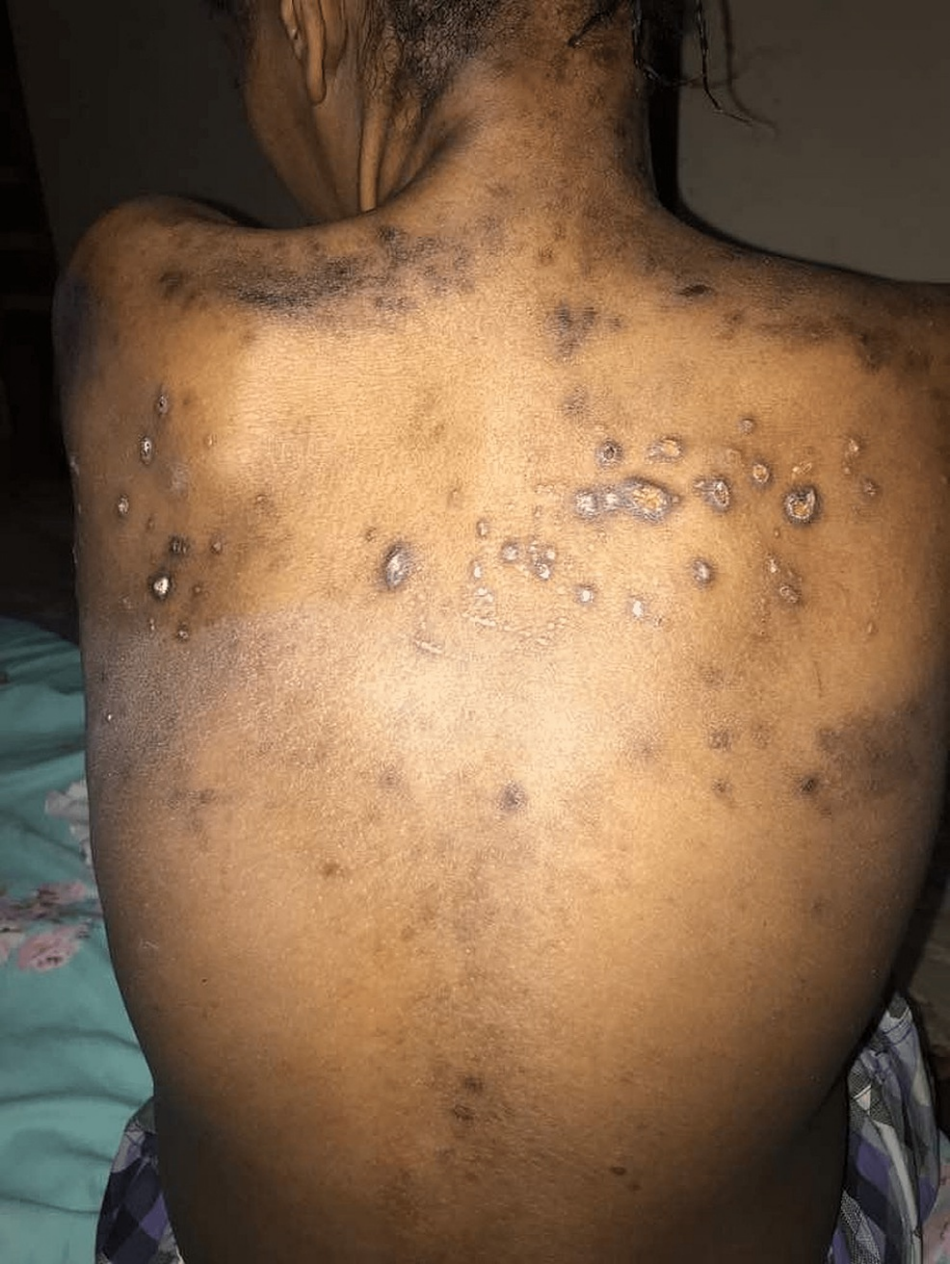
Skin lesions with post-inflammatory hyperpigmentation seen during follow-up eight months after initial diagnosis

She was conscious and alert with intact cognition and had no cerebellar signs or cranial nerve deficits. She had normal motor findings in the upper limbs but exhibited spastic paraparesis with the muscle power of Medical Research Council grade 3+ in both lower limbs. Sensory examination showed anesthesia in the L1 and L2 dermatomes on the right and hypoesthesia up to T3/T4 bilaterally, with intact joint position sensation. Her right-eye vision was normal, but she could only perceive hand movements in the left eye. She had a dilated pupil of 7 mm with a relative afferent pupillary defect and displayed a generalized pale disc with a cup-disc ratio of 0.2 and distinct disc margins. Furthermore, there was asymmetrical suspended anesthesia in all sensory modalities from T1 on the right side to T3 on the left side, extending until L1. Skin examination revealed widespread post-inflammatory hyperpigmentation (Figure 2).

Investigations 

**Figure 3:**
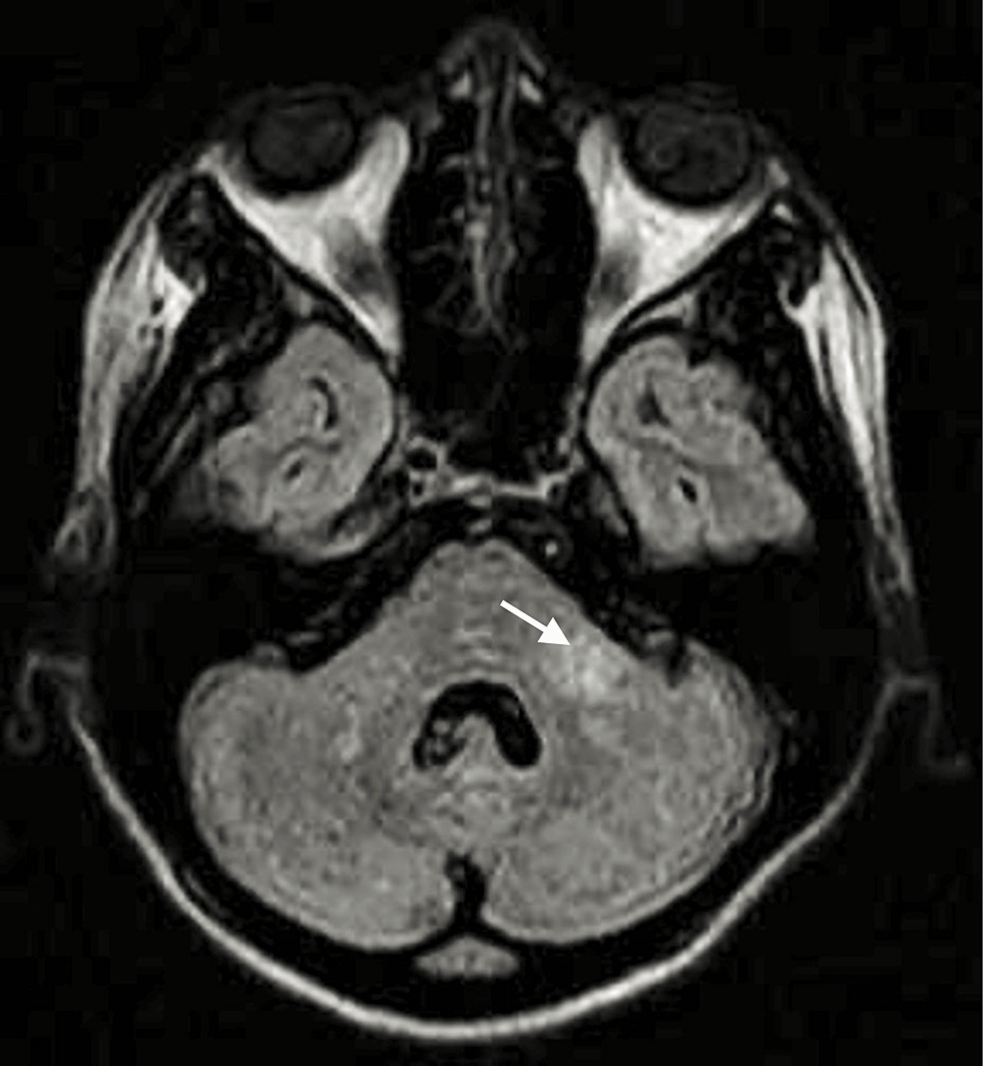
An axial T2-weighted MR image demonstrates hyperintensity (white arrow) in the left cerebellar peduncle

**Figure 4:**
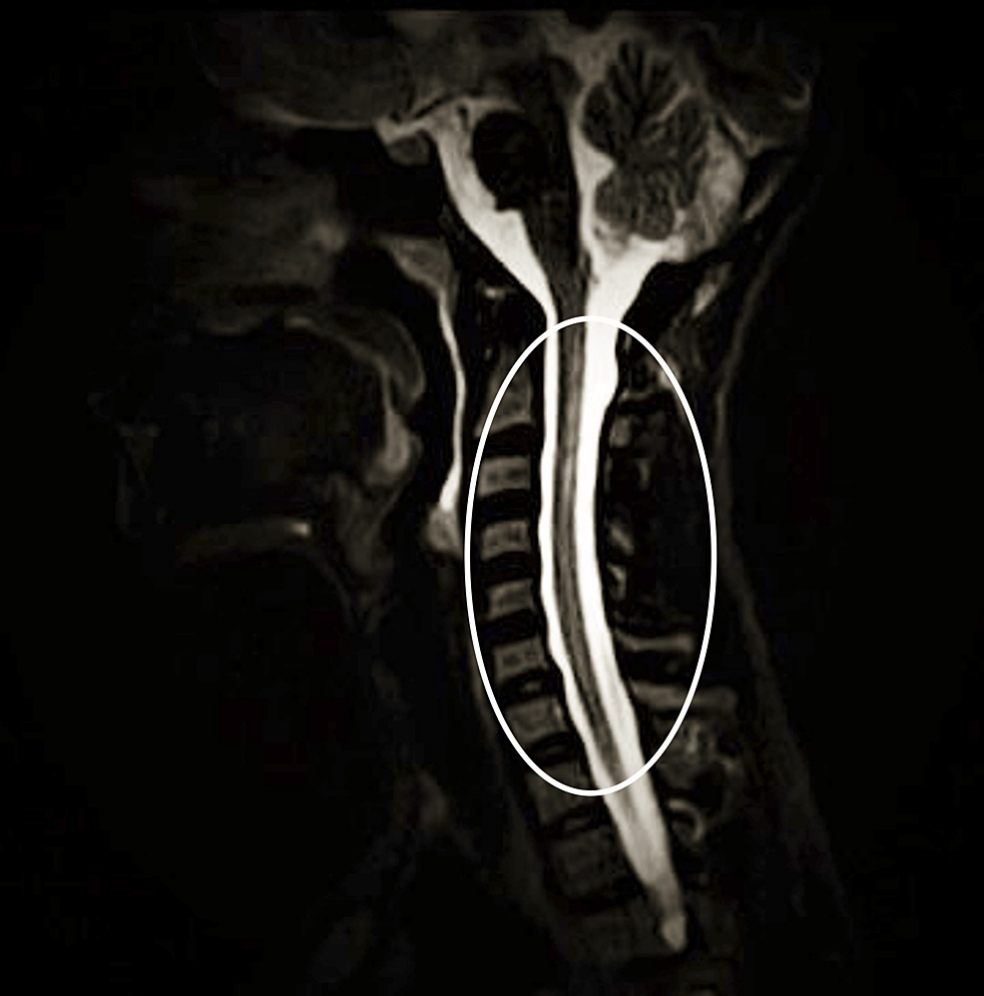
A sagittal T2-weighted MR image demonstrates hyperintensity of the cervical cord

The complete blood count, blood glucose, and renal function tests were within the normal range. Her liver function tests and bilirubin levels were also normal. She had normal chest x-rays, was screened negative for HIV-1, HIV-2, and syphilis, and had a hemoglobin value of 9.3 g/dl. Her urine microscopy showed two to four white blood cells and numerous red blood cells per high-power field, with no growth on urine culture. Repeat urine microscopy showed no red blood cells; thus, the initial result was attributed to contamination from lochia due to poor collection technique. The MRI revealed a non-contrast-enhancing T2 hyper-intense lesion in the left cerebellar peduncle and an intra-medullary non-contrast-enhancing cord (Figures 3-4).

The cerebrospinal fluid aquaporin-4 assay was positive at a 1:320 dilution, and the cerebrospinal oligoclonal band was weakly positive. At this time, she had negative serum double-stranded DNA, negative anti-neutrophil cytoplasmic antibodies, an erythrocyte sedimentation rate of less than 20 mm/hr, and a positive serum anti-nuclear antibody at a 1:80 dilution.

Diagnosis 

A diagnosis of NMOSD was considered using the international consensus diagnostic criteria [1]. The patient had at least one core clinical characteristic (acute myelitis), a positive test for anti-aquaporin-4 IgG (AQP4 IgG), and an exclusion of alternative diagnoses. Other differential diagnoses considered included post-infectious transverse myelitis, generalized tetanus with skin lesions as the site of onset, and intrapartum bilateral nerve compression, and these were systematically ruled out.

Treatment, outcome, and follow-up

After delivery, she commenced oral prednisolone at a dosage of 1 mg/kg, resulting in an improvement in motor and sensory symptoms but no improvement in visual acuity. Oral prednisolone was then discontinued and replaced with azathioprine after 60 days to prevent the potential long-term adverse effects associated with chronic steroid use. Adherence to the medication regimen was assessed based on self-report.

Over the course of several months, she gradually regained motor strength, regained full mobility with no significant disability, and experienced complete resolution of skin lesions. Although she returned to her daily routine, she did not report any improvement in the visual acuity of her left eye, which remained at light perception.

**Figure 5:**
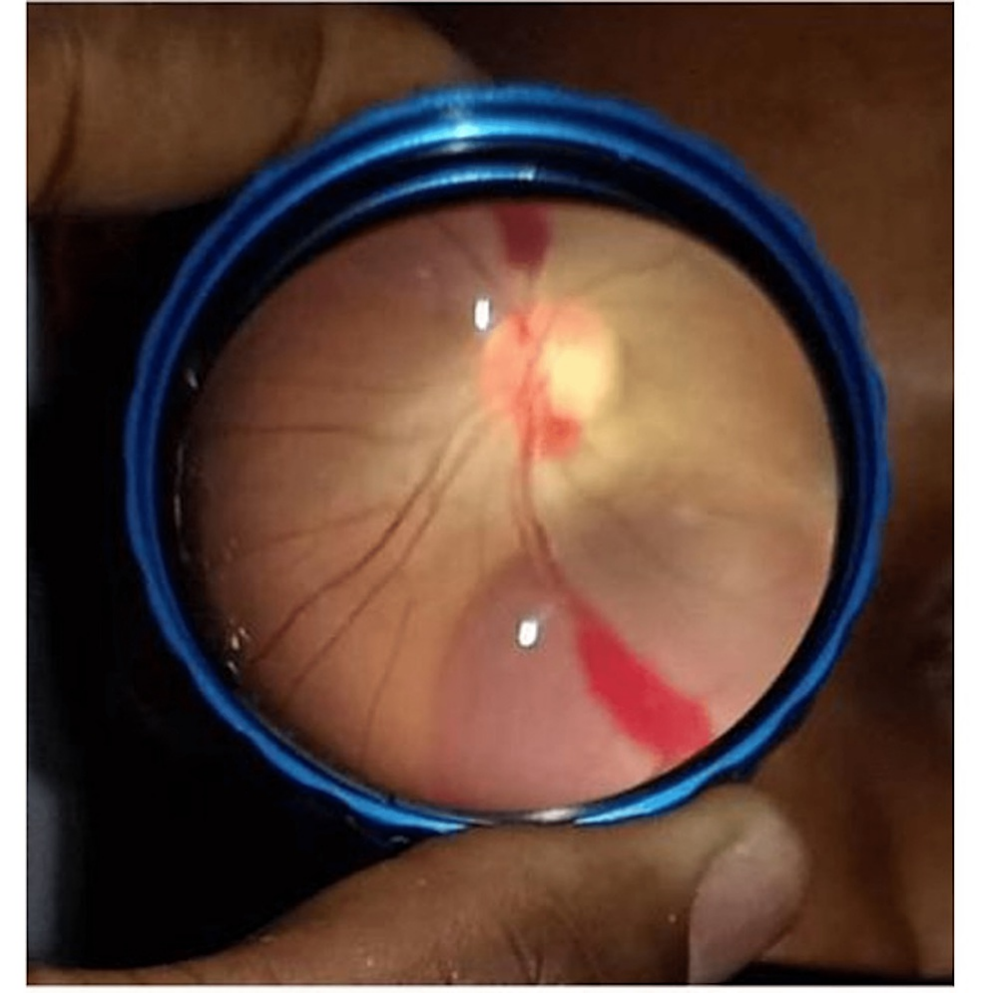
Fundoscopic examination shows pre-retinal hemorrhage with vitreous involvement

Two years after her initial diagnosis, she complained of a sudden and progressive loss of vision in her right eye. The ophthalmologist managed her condition conservatively due to a pre-retinal hemorrhage (Figure 5).

Repeat investigations, including a diabetes screen, baseline blood work-up, clotting profile, erythrocyte sedimentary rate, and anti-neutrophil cytoplasmic antibodies, all returned normal results. Furthermore, a positive pregnancy test prompted further investigations, confirming an unintended pregnancy. However, she chose to undergo a voluntary termination of the pregnancy and refused contraception due to religious beliefs. She was restarted on azathioprine, and subsequent follow-up revealed she regained full vision in her right eye, an aquaporin-4 positive result at a titer of 1:100, normal liver function, and a normal white blood cell count.

**Figure 6:**
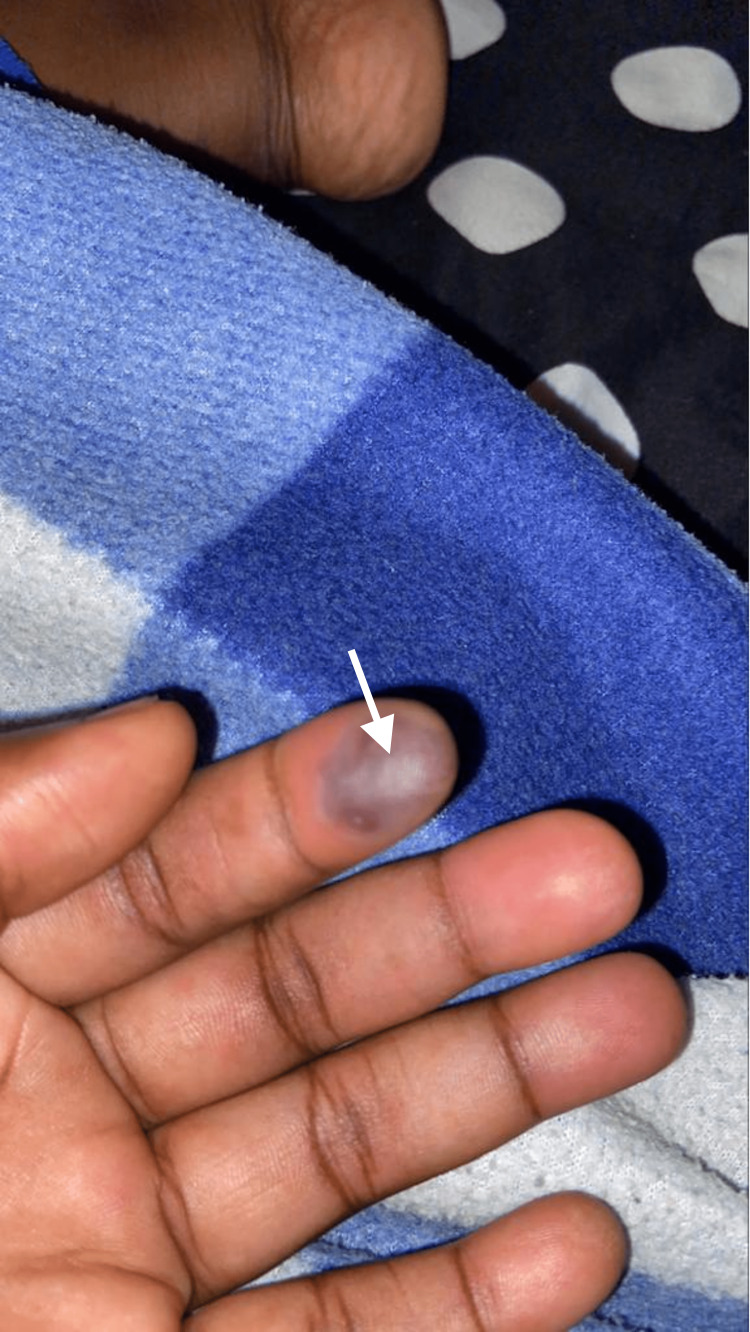
New vasculitic lesions on the pulp of the fingers

Three years later, after the initial diagnosis of NMOSD, she complained of generalized joint pains, malaise, fever, and anorexia. Subsequently, she developed palpable purpuric lesions mainly on the tip of her left index finger, which eventually became ulcers and infarctions (Figure 6).

She was reviewed by dermatologists, and a repeat autoimmune antibody panel was ordered. Her repeat work-up for double-stranded DNA (dsDNA) came out positive. The rheumatology team initiated her treatment with a three-day course of bolus intravenous prednisolone at a dosage of 500 mg per day, followed by a maintenance regimen consisting of low-dose glucocorticoids, hydroxychloroquine, and azathioprine. According to the latest update, she has been in remission for several months and has successfully resumed her regular daily activities.

## Discussion

Our report describes the unusual presentation of a woman with skin manifestations preceding paraparesis and optic neuritis in NMOSD who had an uneventful delivery of a neonate and subsequently seroconverted to a positive antibody for systemic lupus erythematosus (SLE). The clinical and radiological evidence available fulfills the core clinical characteristics of NMOSD [1]. A few concerns might be the absence of contrast enhancement on MRI imaging, likely due to a delay in neuroimaging done many months after symptom onset. Also, the recommended cell-based assay for AQP-4 antibody was not done, an option unavailable in our environment in sub-Saharan Africa. Instead, we utilized the immunoassay detection method. However, the presence of more than one core clinical characteristic, the titer level of aquaporin-4, and the absence of the cerebrospinal fluid (CSF) oligoclonal band strongly support our diagnosis [1]. Regarding the neurologic syndromes that mimic NMOSD, a normal chest x-ray and related clinical findings were used to exclude sarcoidosis, as serum angiotensin-converting enzyme or interleukin-2 receptor levels could not be assessed due to financial limitations. Conventional radiological findings in keeping with multiple sclerosis were absent, and negative retroviral screening and the venereal disease research laboratory (VDRL) test excluded chronic neurotropic infections.

Increasing reports of NMOSD during pregnancy have been published, with pregnancies associated with increased disease activity and more severe disabilities postpartum [3]. Theories behind increased disease activity during pregnancy include up-regulation of aquaporin-4 in the brain during pregnancy and postpartum, expression of aquaporin-4 in the placenta, and the cessation of aquaporin-4 down-regulation in the placenta during pregnancy [6-8]. Our patient experienced heightened disease activity after every conception, with varying degrees of symptom resolution. Interestingly, NMOSD did not affect her fertility or the viability of her pregnancies.

Prior to every paraparesis event, our patient developed intensely pruritic, hyperpigmented macular lesions on her trunk and extremities, which subsequently transformed into papular eruptions accompanied by serous discharge. These healed with post-inflammatory scars. Very few cases of skin lesions accompanying NMOSD have been described in the literature [9, 10]. Although many of these skin lesions are related to overlapping autoimmune diseases [11], our patient developed skin lesions years before the SLE workup became positive and did not satisfy any of the domains in the European League Against Rheumatism (EULAR) criteria at that time [12]. A review of the literature demonstrates that skin lesions in NMOSDs occur via three main mechanisms: neuropathic cutaneous manifestations usually associated with other neuropathic symptoms like anhidrosis, erythema, and pruritus [6-8]; the presence of co-existing autoimmune diseases including dermatomyositis, autoimmune pancytopenia, and dermatitis herpetiformis [13]; and post-viral infectious complications [14], which have been reported to precede disease onset and/or relapse in up to 30% of individuals [15, 16].

Independent risk factors for pregnancy-related attacks include old age at delivery, AQP4-IgG, inadequate treatment during pregnancy, and the postpartum period [5], all of which were present in our case. Increased NMOSD-related disability and relapses during pregnancy and the postpartum period may respond to aggressive management with corticosteroids and immunosuppressants such as azathioprine, which are safely administered during pregnancy and lactation. Studies have shown that patients with AQP4-IgG-positive NMOSD have a high risk of developing pregnancy complications, especially if the disease is active during or just before pregnancy and the patient is not on immunosuppression [4]. The occurrence of retinal hemorrhage in our patient may be linked to NMOSD, considering retinal vasculitis has been reported in patients with NMOSD [17].

Several reports have associated NMOSD with other antibody-mediated diseases, including Sjögren's syndrome and SLE [18]. The precise mechanism leading to the occurrence of NMOSD with comorbid systemic autoimmune conditions remains unclear. A systematic review by Shahmohammadi et al. [19] reported 19 SLE patients who had concurrent NMOSD manifestations. Many of those patients had a positive AQP4 antibody, with the SLE diagnosis preceding NMOSD manifestations in 73.7%. Interestingly, a reverse was seen in our patient, who had a negative antinuclear antibody (ANA) and anti-double-stranded DNA (anti-dsDNA) assay when NMOSD was initially diagnosed but later had seroconversion to a positive result years later during an episodic relapse. The absence of comorbidities associated with SLE during those years contributes to favorable pregnancy outcomes as well.

## Conclusions

In this case report, we have detailed the uncommon clinical trajectory of a female patient who experienced cutaneous lesions prior to the diagnosis of NMOSD during pregnancy, followed by secondary SLE two years after the initial NMOSD diagnosis. The occurrence of cutaneous lesions as clinical manifestations of NMOSD is exceedingly rare. This report also highlights the impact of pregnancy on heightened disease activity. Physicians should maintain a heightened level of suspicion for NMOSD in patients who exhibit recurring skin lesions preceding paraparesis events. In addition, detailed counseling of patients is warranted to prevent permanent visual disability.
